# Outbreak History, Biofilm Formation, and Preventive Measures for Control of *Cronobacter sakazakii* in Infant Formula and Infant Care Settings

**DOI:** 10.3390/microorganisms7030077

**Published:** 2019-03-12

**Authors:** Monica Henry, Aliyar Fouladkhah

**Affiliations:** 1 Public Health Microbiology Laboratory, Tennessee State University, Nashville, TN 37209, USA; mhenry3@my.tnstate.edu; 2 Cooperative Extension Program, Tennessee State University, Nashville, TN 37209, USA

**Keywords:** *Cronobacter sakazakii*, powdered infant formula, *Cronobacter* outbreaks, preventive measures, infant care setting

## Abstract

Previously known as *Enterobacter sakazakii* from 1980 to 2007, *Cronobacter sakazakii* is an opportunistic bacterium that survives and persists in dry and low-moisture environments, such as powdered infant formula. Although *C. sakazakii* causes disease in all age groups, infections caused by this pathogen are particularly fatal in infants born premature and those younger than two months. The pathogen has been isolated from various environments such as powdered infant formula manufacturing facilities, healthcare settings, and domestic environments, increasing the chance of infection through cross-contamination. The current study discusses the outbreak history of *C. sakazakii* and the ability of the microorganism to produce biofilms on biotic and abiotic surfaces. The study further discusses the fate of the pathogen in low-moisture environments, articulates preventive measures for healthcare providers and nursing parents, and delineates interventions that could be utilized in infant formula manufacturing to minimize the risk of contamination with *Cronobacter sakazakii*.

## 1. Introduction

*Cronobacter sakazakii* is a recently classified and an emerging and opportunistic pathogen, found in a number of low-moisture foods including in powdered infant formula. Capable of causing morbidity in all age groups, this pathogen affects neonates and infants leading to life-threatening health complications, such as neonatal meningitis, urinary tract infection, sepsis, and seizures [1,2]. Historically, the pathogen was known as yellow-pigmented *Enterobacter cloacae* [3] until it was reclassified in 1980 as *Enterobacter sakazakii* by Farmer et al. [4,5]. With advancements in identification methods such as partial 16S ribosomal DNA, as well as hsp60 sequencing and polyphasic analyses, the genus undergone further reclassification in recent years [1,4]. Two proposals for defining the new novel genus *Cronobacter* were posited in 2007 and 2008 [4,6], that were further defined in 2012 [7]. The genus *Cronobacter* was derived from the Greek term “Cronos,” a Titan of ancient mythology who swallowed his infants when they were born, in fear of being replaced by them [5]. The species epithet *sakazakii*, was proposed by Farmer et al. in 1980, in honor of the Japanese microbiologist, Riichi Sakazaki (1920–2002), a bacterial taxonomist also involved in nomenclature development of this pathogen [5,8].

*C. sakazakii* is a peritrichously flagellated, rod-shaped, and non-spore-forming pathogen. It is recognized as facultative anaerobic where its preferable growth is without oxygen presence but can grow with a small amount of oxygen. The growth temperature range is 6–45 °C with an optimum multiplication temperature of 37–43 °C. It can also survive low-moisture environments, such as infant formula, with a water activity of 0.30 to 0.83 for up to 12 months [9]. Ranging from 9–44%, *C. sakazakii* can be found in environmental samples from domestic and manufacturing facilities [10]. Thermal resistance can play a major part in the survival rate of *C. sakazakii*. Lukewarm water with temperature ranging from 52–58 °C has been confirmed to reduce the pathogen in reconstituted powdered infant formula [11]. *C. sakazakii* can be cultured on tryptic soy agar showing a distinct morphology of yellow-pigmented colonies. The vast majority of reported cases worldwide are from the United States, France, UK, Belgium, Philippines, Brazil, Israel, Spain, Hungary, Japan, Mexico, China, and Switzerland [1]. It is noteworthy that recent studies indicate that non-*sakazakii* species of *Cronobacter* including *malonaticus*, *turicensis*, *universalis*, *dublinensis*, *muytjensii*, and *condimenti*, could potentially cause morbidity and life-threatening complications in infants and adults [12,13]. Except for *C. condimenti* that has not been involved in any documented clinical episode, the other six species have clinical significance, with *C. sakazakii* and *C. malonaticus* as the major pathogenic species of public health concern followed by *C. turicensis*, *C. universalis*, *C. muytjensii*, and *C. dublinensis* [14,15,16]. As further delineated in Section 3.1, unlike vast majority of foodborne pathogens of public health concern, pathogenic species of *Cronobacter* are currently not part of the notifiable disease surveillance systems in nearly all public health infrastructures of North America and European Union, thus, the true epidemiological picture of these pathogens will continue to be unknown. Currently, identification of *Cronobacter* species can only be made through the use of species-specific PCR analyses or by whole genome sequencing.

## 2. Outbreak and Sporadic Episodes

*C. sakazakii* is an emerging pathogen in neonates and infants that was first known internationally before being recognized in the United States. In the late 1920s, there had been a report of “yellow-pigment coliforms” by Pangalos as the first published information on *Cronobacter* species from a case of septicemia [17]. Fast forwarding to the 1950s, strains from potable and/or river water samples from Metropolitan Water Board in London, submitted to England’s National Collection of Type Cultures, seem to have similar physiological traits of possibly *Cronobacter* species. Nevertheless, between 1958 and 2016, there are approximately eight countries which reported cases of *C. sakazakii* suggesting that its reemergence and increased prevalence is reflective of its increased public health concern [5]. Here are listings of outbreaks in chronological order from the first documented case of *C. sakazakii* outbreak to the current. A summary of these outbreaks was also uploaded and is available in a public repository that can be accessed at https://doi.org/10.7910/DVN/TZ5PV9 (accessed on 21 February 2019).

As delineated earlier, our understanding of pathogenic *Cronobacter* species have been subject of redefinitions in recent years [14,15,16]. Until the correct identification of clinical isolates of *Cronobacter* species is achieved, the epidemiology of infections caused by these pathogens will always be lacking.

### 2.1. 1958: England

In 1958, there were two reported cases of neonatal meningitis at the Osterhills Hospital in England who died within two days apart. Patient 1 was a male, born on May 29, 1958. He had an average birth weight of 3034 g and was born after 38 weeks of gestation. After 10 days of life, the infant was discharged from the hospital but was quickly readmitted the next day after signs of grunting, jaundice, and loss of appetite. Samples were taken from the brain, cerebrospinal fluid, bronchus, urine, and blood where *Enterobacter cloacae* (reclassified in later years to *Enterobacter sakazakii* [18]) was isolated and a diagnosis of meningitis was confirmed. Intramuscular injection of oxytetracycline was given to the patient. However, within 48 h, the patient died.

Patient 2 was a female, born on June 5th, 1958, with her twin brother. After an emergency cesarean section, the newborns were premature after 32 weeks of gestation with the patient and her brother weighing 2013 g and 1191 g, respectively. The brother began to show signs of good progression over five days, however, the patient did not. On day 5 of life, the patient had immediate signs of collapsing cerebral, jaundice and an urticarial rash. Samples from bronchus, liver, marrow, and spleen were taken, but hours later the patient died.

Both of the neonates had similar findings in the necropsy report, one including abnormalities in the brain which may lead to the presence of meningitis. Pertaining to the respiratory tract, patient 1 had no evidence of inflammation unlike patient 2 who contained scanty yellow fluids with consolidated lungs. The strains that were isolated from both neonates were identical and reported as yellow-pigmented Coliform. Being abnormal that patient 2 twin brother was not affected with the pathogen given they were nursed in the same environment, had the same treatments, and shared the same nurse with expectations of using different incubators [19]. Due to lack of microbiological, epidemiological, and bioinformatic evidence, the true source of infection is undetermined in this historic outbreak.

### 2.2. 1965: Denmark

A case is described of neonatal meningitis complicated by brain abscess and hydrocephalus. The etiological agent was an uncommon *Enterobacter* morphologically similar to a strain isolated from the spinal fluid in two cases of neonatal meningitis in 1961 at St. Albans, England [20].

### 2.3. 1979: Macon, Georgia

The first documented case for *C. sakazakii* in the United States of America was at The Medical Center of Georgia, Macon, Georgia in 1979. A male infant who was born healthy was fed on nursery routine formula feedings and was only 30 g lesser than normal birth weight when he was discharged after four days of life. On day 6 of life, the patient became irritable, eating less, and was coming down with a fever. After the temperature was taken at 38.9 °C, the patient was hospitalized for further testing. The patient had a normal urinalysis, platelet count, and umbilicus had no signs of infections. What seemed to be abnormal was the high heart rate of 192 bpm, low leukocyte count, and maintained elevated axillary temperature. Diagnosed with possible sepsis, the patient’s blood samples were then collected and tested positive for *C. sakazakii*. A combination of injections of ampicillin (75 mg every 12 h) and gentamycin (7.5 mg every 12 h) were given to the patient and blood samples were taken again. After seeing that the blood sample isolate was susceptible to ampicillin, gentamycin was discontinued. After six days of being in the hospital and two weeks of age, the dosage of ampicillin increased to 100 mg every 8 h. Blood samples were taken after a week of the new treatment and no *C. sakazakii* could be detected. The patient seemed to be doing well and after ten more days of therapy, ampicillin was discontinued and he was observed for another day. The patient was discharged from the hospital and later came back for a two-month check-up with reported normal development, weight of 5120 g, and no signs of a *C. sakazakii* infection [21].

### 2.4. 1977–1981: Netherlands

Eight infants were infected with *C. sakazakii* over the timeline of four years from 1977 to 1981. This epidemic was the largest in the Netherlands to be reported. Five of the patients (1–5) were admitted into the same hospital (A). Two of the eight patients (6 and 7) were at different hospitals (B and C) at birth and transferred to the same initial hospital when developing symptoms of illness (D). One patient (8) was at another hospital not related to the rest of the patients (E). Patients 6–8 were in the same area in another part of the country from the general hospital of patients 1–5. Out of the eight cases, only two patients, patient 1 and 8, survived [22].

#### 2.4.1. Hospital A

Patient 1 was a male that was in good condition until he started exhibiting complications on day 5 of life in September of 1977. He was born prematurely with a weight of 2830 g after 36 weeks of gestation. Leading up to day 5 of life, the patient’s temperature began to rise to 38.2 °C which is considered as fever in infants. Along with the raising of the temperature, leukocyte counts were 5500/mm^3^ and protein concentration was low from samples taken from the cerebrospinal fluid (CSF). The patient began treatment with ampicillin and kanamycin for 48 h. After a second CSF sample, leukocyte counts decreased while protein and glucose concentrations were still low and the patient’s temperature still elevated at 39 ºC. As a new treatment, gentamicin was given to the patient for an additional 15 days and he recovered with a low leukocyte count and high protein and glucose concentration. However, the patient was diagnosed with a severed neurological sensory development upon recovery [22].

Patient 2 was a female in good condition until she started to show symptoms on day 3 of life in April of 1979. Born with a weight of 2400 g after 39 weeks of gestation. Antibiotics were given to the patient with a combination of ampicillin and kanamycin and no progress was made. Gentamicin was then given and still no progress was made. The patient did not survive the infection [22].

Patient 3 was a female in good condition until day 3 of life. Born with a weight of 1670 g after 32 weeks of gestation, the patient was given ampicillin and gentamicin. Patient 4 was a male that was in good condition until day 4 of life. Born with a weight of 1900 g after 32 weeks of gestation, this patient was given ampicillin and gentamicin. Patient 5 was a female that was in good condition until she started to exhibit complications on day 5 of life. Born with a weight of 2690 g after 38 (Full term) weeks of gestation, the patient also received ampicillin and gentamicin [22].

#### 2.4.2. Hospital D

Patient 6 was a male that was in good condition until the day 5 of life in February of 1978. On day 5, the patient was transferred to this hospital, he was born with the weight of 2085 g after 38 weeks of gestation. Chloramphenicol and gentamicin were given to the patient. Patient 7 was a female that was in good condition until she started exhibiting complications on day 5 of life in September of 1979. Patient 7 was also transferred to hospital D. She was born prematurely with a weight of 1370 g. Two antibiotic regimens were administered for the patient, ampicillin and gentamicin, as well as chloramphenicol and gentamicin [22].

#### 2.4.3. Hospital E

Patient 8 was a female that was in good condition until exhibiting symptoms on day 9 of life in April of 1979. Born weighing 850 g after 30 weeks of gestation. A combination of ampicillin and gentamicin were given to the patient, she later survived and was diagnosed with severed neurological sensory development [22].

### 2.5. 1980: Indianapolis, Indiana

Unlike previously documented episodes that the affected patients were only few days old, this case is the first documented case where an infant was older than one month of life and did not develop symptoms associated with *C. sakazakii* infection during the hospitalization or shortly after. A female after five weeks of age develop a fever and seizure episodes was admitted to a hospital in Indianapolis, Indiana. A cerebrospinal fluid sample (CSF) exhibited high leukocyte count (15,600 per mm^3^), a low protein concentration (295 mg/dl), and very-low glucose concentration (4 mg/dl). A combination of ampicillin (400 mg/kg every 24h) and chloramphenicol (100 mg/kg every 24 h) had begun. *C. sakazakii* was isolated from the CSF samples and treatments were continued with only ampicillin. After six days of therapy, *C. sakazakii* was continuing to proliferate in the serosanguinous fluid and gentamicin (7.5 mg/kg every 24 h) was added into the treatment. On day 15, *C. sakazakii* continued to persist and computed tomography (CT) showed massive ventricular dilation. The patient was transferred to a hospital for more advanced observation and assistance, the combination of ampicillin and gentamycin were continued. After the last positive sample of the pathogen from the patient’s ventricular fluid, ampicillin and gentamicin were continued to be administered for 21 days. Ventricular fluid was then tested negative 24 h after discontinuation of the antibiotics. After two months, the patient was discharged, however, the circumference of the head continued to increase and developing skills were severely delayed [23].

### 2.6. 1981: Oklahoma City, Oklahoma

A male born from a healthy delivery was admitted into the hospital at five weeks of age from symptoms of fever, grunting, and fatigue. The temperature was taken with a high reading of 39.2 °C and heart rate of 180 bpm. Neurological symptoms included the absence of rooting and sucking reflexes and incomplete Moro reflex. The CSF sample exhibited leukocyte count 2871/mm^3^, glucose concentration of 46 mg/dl and protein concentration of 168 mg/dl. Along with CSF, blood and urine samples were taken and sub-cultured to sheep blood agar and *C. sakazakii* was detected. Gentamicin and ampicillin were given to the patient until the test showed the pathogen was susceptible to ampicillin then gentamicin was discontinued. After 14 days of treatment with ampicillin, the patient was discharged in good condition [24].

### 2.7. 1982: Greece

*C. sakazakii* was first isolated in Greece in 1982 from the fecal samples of two thalassaemic children. Limited pieces of information were available in the reviewed citation about the cases [25].

### 2.8. 1984: Greece

A neonatal intensive care unit in Greece had 11 neonate-associated *C. sakazakii* infections reported from September 10 to October 17, 1984. The neonates had swabs from the throat and rectum on first or second day after admission and again after three to four days, and follow-up weekly sampling thereafter. After strains were plated on blood agar, MacConkey’s, Chapman’s, and Sabouraud’s agar; twenty-eight strains were identified as *C. sakazakii*. Along with the neonates being tested, environmental surfaces, medical fluids in the unit, and 77 fingertips of the staff were also tested for the presence of *C. sakazakii*. Isolates of *C. sakazakii* was not found on abiotic environmental surfaces, medical fluids, nor the staff. Out of the 11 patients, seven survived [25].

### 2.9. 1986–1987: Reykjavik, Iceland

Three cases were reported in Reykjavik, Iceland of neonates contracted with *C. sakazakii* in 1986–1987 [26].

Case 1: A male born on March 18, 1986, after 36 weeks of gestation. He had a birth weight of 3144 g and appeared to be healthy with feeding of breast milk and powdered infant formula. On day 5 of life, the patient’s health began to deteriorate and his spinal fluid was taken for microbiological analyses. *C. sakazakii* was isolated from the cultured spinal fluid sample and blood. Treatments of ampicillin and gentamicin began immediately along with cefuroxime within 12 h. After two weeks, cultures of *C. sakazakii* were still positive from ventricular fluids and chloramphenicol was added to the treatments for two months. The patient was discharged from the hospital at three months of age with his mental and physical development considered “markedly impaired”. At the age of two years, the patient was diagnosed with severed neurological sensory development and quadriplegic [26].

Case 2: A male born December 14, 1986, with a weight of 2508 g had Down’s syndrome. He was orally fed reconstituted powdered infant formula hours after his anoplasty surgery and exhibited no health complications until day 5 of life when he started to eat poorly. Patient’s health began to deteriorate quickly and electrocardiograms and ultrasonograms were taken. The *C. sakazakii* infection was confirmed in the spinal fluid. Treatments of ampicillin and cefotaxime were not successful and the patient did not survive. Meningitis was confirmed from the autopsy [26].

Case 3: A male of a twin was born after 38 weeks of gestation on January 6, 1987, with a weight of 3308 g, reportedly healthy and was feeding on breast milk and reconstituted powdered infant formula until day 5. On day 6, he had a fever and his health was deteriorating quickly. Cerebrospinal fluid (CSF) samples were taken with a high leukocyte count and *C. sakazakii* was isolated but the blood was negative. Ampicillin and cefotaxime were started and health improvements were shown. The second testing of CSF was negative for *C. sakazakii* and antibiotics discontinued after three weeks. He was discharged after one month but CT scans did show cystic cavity in the left frontal lobe. He exhibited seizure disorder and delays in developmental areas [26].

### 2.10. 1988: USA

For the reported two cases, limited pieces of information were provided about the patients’ progress in the cited literature [27].

### 2.11. 1988: Memphis, Tennessee, USA

In March of 1988 at a neonatal intensive care unit in Memphis, Tennessee four infants, two with bacteremia, another with a urinary tract infection, and one with bloody diarrhea had isolates of *C. sakazakii*. It was found that all four infants were fed from the same infant formula batch used in a blender. The staff’s cleaning procedure was cleaning the blender with tap water and handwashing agents, but after being cultured a heavy growth of *C. sakazakii* was found on the preparation equipment. The blender was discontinued for use until it was sterilized, after being sterilized no cultures of *C. sakazakii* was detected [28].

### 2.12. 1989: Porto, Portugal

At a hospital in Porto, Portugal, there were 187 cases of meningitis where 15 patients were neonatal, 79 infants, and 93 between the ages of 1–14 years. Among these cases, 15 patients died and two out of the fifteen were infected with *C. sakazakii*. They both were neonates with infant formula consumption [29].

### 2.13. 1990: Maryland, USA

One case was reported in the literature with limited pieces of information about the patient’s prognosis and survival [27].

### 2.14. 1990: Ohio

At a children’s hospital in Cincinnati, Ohio, a 2520 g male was born of 35 weeks, exhibiting symptoms of poor feeding, apnea, and bradycardia after day 2 of life. Blood samples were taken from the patient and *C. sakazakii* was found. Ampicillin and cefotaxime were administered on day 4, CT scans were taken and the patient was shown to have increased tension on the left side of the brain. After three weeks of treatment, the patient showed no sign of inflammation and was discharged on day 28. About two weeks after discharge, the patient, with reported consumption of infant formula, was readmitted into the hospital with symptoms of poor feeding and fever. He was diagnosed with meningitis and the antibiotics of ampicillin and cefotaxime were started again. After the treatment cerebrospinal fluid results came back negative but after another CT scan was taken, an abscess was found in the brain. The cyst was drained (did not tested positive for *C. sakazakii*), and the patient had a resolving cerebral infraction [30].

### 2.15. 1993–1998: Jerusalem, Israel

At a hospital in Jerusalem from 1993–1998, four cases of neonates were infected with *C. sakazakii*, the bacterium was additionally isolated from a blender used to mix and prepare infant formula [31].

Case 1: In 1993, a neonatal born in a full term and fed infant formula in the hospital tested positive for *C. sakazakii* [31].

Case 2: In 1995, a healthy female born by caesarian section after 36 weeks of gestation developed conjunctivitis, she was fed infant formula and tested positive for *C. sakazakii* [31].

Case 3: In 1997, *C. sakazakii* was found in a 6-year old boy from bone marrow transplantation for lymphoblastic leukemia [31]. 

Case 4: In 1998, a vaginally-delivered, full term female infant was admitted with a diagnosis of meningitis which *C. sakazakii* was cultured from the infant’s CSF [31].

### 2.16. 1994: France

Thirteen cases were reported in the cited literature with limited pieces of information about the patients’ prognosis and survival [32].

### 2.17. 1998: Brussels, Belgium

Between June and July of 1998, 12 cases of neonates in neonatal intensive care unit of a Hospital were being contracted with *C. sakazakii*. This is the largest documented case in the history of this pathogen infecting neonates. All 12 patients had a birth weight of <2000 g and were orally fed a powdered infant formula before the development of neonatal necrotizing enterocolitis. Only two patients, male twins, died from this outbreak [33].

### 2.18. 1999–2000: Jerusalem, Israel

In the same hospital as the previous case in Jerusalem, 2 patients contracted *C. sakazakii* in 1999 and 2000. Patient 1 was a female born at 27 weeks of 620 g in December of 1999. On the ninth day of life, the infant was diagnosed with *C. sakazakii* infection after being fed with infant formula. The patient responded well to cefotaxime and survived. Patient 2 was a female of 36 weeks gestation with a weight of 2155 g. She was delivered by caesarian section because of fetal distress. Delivered 3 weeks after patient 1 in January 2000, *C. sakazakii* was cultured from CSF on day 4 of life. Treatments of cefotaxime and gentamicin were given but severe damage occurred in the brain. The patient survived and after three months was discharged with neurological problems. The infant was fed infant formula before being infected with *C. sakazakii* [31].

### 2.19. 2000: North Carolina, USA

One case was reported in the cited literature with limited information about patient’s survival and prognosis [27].

### 2.20. 2001: Knoxville, Tennessee, USA

In 2001, a neonate was born of 1276 g through a caesarean section at 33.5 weeks. Patient being underweight at birth, intensive care was needed for the infant. Along with the low weight, the patient had a fever, tachycardia, decreased vascular perfusion, and neurologic abnormalities at 11 days. After another nine days, the patient passed away with a trace of *C. sakazakii*. This patient and 49 others were microbiologically screened in addition to obtaining environmental samples from the infant formula and preparation area. By the end of the screening, it was determined that the infant that died was infected through the powdered infant formula feed and no other patient was infected [34]. It is noteworthy that this outbreak is the first documented incidence of a manufactured lot of powdered infant formula being intrinsically contaminated. Thus, this outbreak epidemiologically linked powdered infant formula with *Cronobacter*.

### 2.21. 2002: Wisconsin, USA

One case was reported in the cited literature with limited pieces of information about patient’s survival and prognosis [27].

### 2.22. 2002: Chandigarh, India

A female born at 34 weeks of gestation, weighing 1400 g, was re-admitted to the hospital on July 2002. The neonate was put on oral rehydration due to respiratory problems. After day 5 of life, the infant was put on a ventilator and developed sepsis with meningitis after development of grunting, episodic apnoea, chest retraction, and tachypnoea. Cerebrospinal fluid samples exhibited a high protein concentration, low glucose concentration, and elevated leukocyte count. The infant started an antibiotics chemotherapy of ciprofloxacin and netilmicin after a positive blood culture for *C. sakazakii*. The isolate showed resistance to ciprofloxacin, cefotaxime, and ceftazidime and sensitivity to gentamicin, amikacin, netilmicin, and co-trimoxazole. The infant did not survive the infection [35].

### 2.23. 2003: USA

Six neonatal cases with infant formula consumption were reported in the literature with limited information about the diagnosis and survival of the cases [27].

### 2.24. 2004.: France

On October 25th, 2004, and December 7, 2004, from two different hospitals, two neonatal cases were documented with *C. sakazakii* infection. Another two cases were identified at two separate locations by a regional public health surveillance system in early December. In all episodes, there were four cases and four hospitals involving neonates being contracted with *C. sakazakii*. Two of the four patients died [36].

### 2.25. 2004: USA

Two cases were reported in the cited literature with limited pieces of information about the patients’ diagnosis and prognosis [27].

### 2.26. 2005: USA

Two cases were reported in the referenced study with limited information about the cases’ medical history, prognosis, and survival [27].

### 2.27. 2006: Chandigarh, India

In the same hospital as the last case in India, another case of *C. sakazakii* occurred four years later. A two-month female infant was on breastfeed and was admitted to the hospital in July 2006 with a cough and respiratory distress. Prior to the patient’s admission, the mother had hypertension and diabetes that required insulin; this caused the infant to suffer from jaundice on day 3 of life. After three days of being in the hospital, the infant developed sepsis and was transferred to pediatric intensive care. Blood cultured positive for *C. sakazakii* and was resistant to many of the antibiotics while exhibited sensitivity to the ceftriaxone-sulbactam combination. Treatment with ceftriaxone-sulbactam started with vancomycin, initially for five days. After availability of susceptibility data, only ceftriaxone-sulbactam administration was continued for additional two weeks and stopped when the blood culture was negative for *C. sakazakii*. The infant was discharged and reported afebrile [35].

### 2.28. 2007: Bilbao, Spain

A healthy male born 31 weeks and weighed 1715 g until day 3 of life when exhibited poor feeding. On day 5 of life, the patient was diagnosed with sepsis and was on a combination of ceftazidime and vancomycin. After the tenth day of treatment, an improvement was observed in clinical analytics. The patient was fed breast milk for the duration of the hospital and after 24 days, physical examination, serial brain scans, and psychomotor development were normal [37].

### 2.29. 2010: Queretaro, Mexico

In 2010, two infants were infected by *C. sakazakii* in a hospital in Queretaro, Mexico. The infants who were fed infant formula developed bloody diarrhea. Antibiotics (cefotaxime and vancomycin for case 1 and clindamycin and amikacin for case 2) were given and the two patients recovered [38].

### 2.30. 2011: Missouri, Florida, Oklahoma, and Illinois, USA

In 2011, four states in the United States (Missouri, Florida, Oklahoma, and Illinois) had cases of *C. sakazakii* infections. In Missouri, a 10-day old infant died from *C. sakazakii*, the bacterium later found in the infant formula, bottle of nursery water, and the serving container. Immediately, a major retailer recalled that brand of powdered infant formula from its stores nationwide on December 22, 2011. A leading regulatory agency of the country tested factory-sealed containers of the formula and nursery water of the same batch and no cultures of *C. sakazakii* were found. In Florida, an infant died of *C. sakazakii* infection, however, the strain of that case could not be obtained, nor in the case in Oklahoma. The strains from the Missouri and Illinois cases were gathered but it was not genetically related to that which was isolated from the reconstituted powdered infant formula and nursery water [39].

### 2.31. 2015: Sydney, Australia

In 2015, a male infant was born prematurely after 27 weeks of gestation without any signs of health complications. However, patient’s health suddenly deteriorated on day 10 of life and blood cultures tested positive for *C. sakazakii*. After an unsuccessful antibiotic treatment with meropenem, patient was redirected to palliation after discussion with parents and died at 11 days after birth. *C. sakazakii* was isolated from breast milk expressed by a handheld breast pump that had not been properly sterilized before use [40]. The isolates from patient’s blood and the expressed milk were identical based on bioinformatic evidence derived from whole genome sequencing of the patient and breast milk isolates [40].

### 2.32. 2016: Pennsylvania, USA

In April 2016, a female born at 26 weeks of gestation and weight of 1405 g was healthy until 21 days of life when she was diagnosed with sepsis. Samples taken from the cerebrospinal fluid and blood showed *C. sakazakii* presence. Treatments of ampicillin and cefepime were given however, seizures developed and the brain had liquefaction necrosis. The infant did not receive any powdered infant formula, however, pasteurized donor human milk and expressed maternal milk were given during the first week after birth. *C. sakazakii* was isolated from the breast pump kit and the kitchen sink drain from the mother’s home [41].

It is noteworthy that in addition to the above-referenced episodes of infant morbidity and mortality associated with pathogenic *Cronobacter*, a study of 2012 [42], have summarized 68 cases from 1958 to 2003 and 30 cases belonging to 2004 to 2010. The study had accumulated the information based on personal communications, health records from the U.S. Centers for Disease Control and Prevention, the U.S. Food and Drug Administration and the World Health Organization, and published records. Since patients’ specific prognosis and condition were not provided, those studies are not included in the current list of outbreaks. The 2012 study concludes that *Cronobacter* can infect both healthy term and hospitalized preterm neonates, and further recommends use of ready-to-feed formula for infants <2 months [42]. Differences among various types of infant formula are presented in Section 3.2 of the current study.

## 3. Recommendations for Parents and Caregivers

### 3.1. Vulnerable Population

*C. sakazakii* is a pathogen found primarily in dry and dehydrated food vehicles with low water activity, such as herbal teas, starches, and most concerning in powdered infant formula. The bacterium usually causes no health complications in adults, while could lead to sepsis, severe meningitis, and possible deaths in infants that are less than 12 months old [2]. Centers for Disease Control and Prevention estimates that four to six infants are infected with *C. sakazakii* each year in the United States. Although Minnesota Department of Public Health requires reporting of *C. sakazakii* in infants under one year of age within one business day of positive test results [43], in other states, *C. sakazakii* is not a reportable disease in infants nor adults, therefore almost certainly this is an underreported infection in the United States [2]. In neonates and infants, the symptoms start with fever and poor feeding, crying, and very low energy. In some severe cases, seizures, brain abscess, and high leukocyte counts occur, that could lead to long-lasting brain problems such as severed neurological sensory development [2]. The disease is more prevalent in premature infants that have low birth weights and possibly diagnosed with malnutrition such as low iron. Though very rare, *C. sakazakii* could also infect people of all ages, it is typically more severe for the elderly. Immunocompromised individuals including cancer patients, those having HIV and organ transplants are the adults who are more susceptible to *C. sakazakii* infection. Diagnosis of *C. sakazakii* infection is through blood sampling and usually followed by testing the cerebrospinal fluid for leukocyte count, glucose and protein concentration, and other special testing with the brain [2].

### 3.2. Infant Formula Manufacturing and Common Exposure Routes of C. sakazakii

The transmission of *C. sakazakii* is widely associated with reconstituted powdered infant formula. Micronutrients used in the formulation of powdered infant formula are heat-labile, therefore, it must be added after the pasteurization/heat treatment to keep the nutritional value in compliance with regulatory standards [10]. Infant formula is designed as a substitution for breastfeeding to mimic the nutritional properties of breast milk. Despite assumptions of many new parents, due to considerable compositional differences, cow’s milk could not be utilized for feeding newborns, making the infant formula the only practical alternative to breastfeeding. There are three commercially available sources of infant formula: (1) Powdered formula, the least expensive and most popular, and must be mixed with water; (2) liquid concentrate, which must be mixed with an equal amount of water; and (3) ready-to-feed products that are the most expensive products and require no mixing with additional liquids [44]. 

The manufacturing processing of each of these infant formulas is different. For powdered infant formula, it undergoes two processes, dry-blend, and wet-blend spray drying. Dry blending to produce powdered infant formula is practiced by many firms in at least 40–50 processing plants worldwide [45]. The process begins with ingredients that have been tested for microbiological contamination and blended in large batches until nutrients/ingredients are distributed uniformly in a batch. It is then passed through sifters to remove oversized particles and other extraneous materials. The sifted product is then transferred to bags, totes, or lined fiberboard drums for storage. For canning the powdered infant formula prior to release to the market, it is flushed with inert gas, sealed, labeled, coded, and packaged into cartons. The packed product then undergoes a final check for microbiological contaminants. The ingredients that are used for this processing method are in dehydrated powdered form tested by the supplier(s). Since this process does not require extensive thermal processing, it is very critical that microbiological testing is conducted as well as working with reputable suppliers with validated food safety management plans in place [45]. Microbiological contamination might be present in low amounts, distributed heterogeneously and, thus, may be difficult to detect in random lot testing alone. 

Wet blend-spray drying is another method to produce powdered infant formula. This process also begins with ingredients from suppliers that have been tested then it goes through pasteurization where the destruction of microbial cells will occur due to a thermal treatment in a relatively short amount of time. Next is homogenization, where the size of fat and oil particles are being reduced to have a uniform mixture, some companies may do this step before pasteurization [45]. Since powdered infant formula is designed to mimic the nutritional properties of breast milk, heat-sensitive micronutrients, such as vitamins, amino acids, and fatty acids, are then added after pasteurization where they will otherwise become inactivated/denatured by intensive heat. The mixture may now pass through an evaporator that is heated up to 62–77 °C and transferred through a high-pressure pump to spray dryer nozzles or cooled for storage, then reheated, and pumped directly to the spray dryer. As the mixture passes through the nozzles of the spray dryer, the water is evaporated and dry powder is created at the bottom of the spray dryer ranging in temperature typically from 73–79 °C. It is then cooled by a stream of chilled filtered air and passed through a sifter for packaging. It is also checked a final time for microbiological contaminants through random sampling. One disadvantage with wet blending followed spray drying is that it contains water in its processing and has a higher chance of the proliferation of pathogenic or spoilage bacteria. Liquid concentrate and ready-to-feed infant formula are similarly processed, then pasteurized using ultra-high temperatures (UHT) [46]. For these products, in short, first, the ingredients from suppliers are mixed together then skim milk is added at 60 °C, then fats, oils, and emulsifiers. During the formulation of this mixture, minerals, vitamins, and stabilizing gums are then added at various points due to sensitivity to heat. Next is pasteurization through heat exchange plates at a high temperature, typically from 85–94 °C for a short time of 30 seconds [46]. Homogenization is next for a uniform mixture followed by standardization of the correct parameters for pH, fat content, vitamins, and minerals. The last stage is packaging into containers followed by sterilization with heat. The main difference between liquid concentrate formulas and the ready-to-feed formulas is the amount of water required to be added during the preparation of the product. Liquid concentrated and ready-to-feed infant formulas are safer to use due to the existence of high-heat pasteurization and/or sterilization relative to powdered infant formula, however, it is typically more expensive [46].

In summary, addition of heat sensitive ingredients to improve nutritional value of the formula and meeting the strict nutritional regulatory requirement and difficulties in pasteurizing/sterilizing a product in powdered form are main concerns for safe production of infant formula. Other transmission routes of *C. sakazakii* to infants can be associated with the preparation methods at home or care settings. *C. sakazakii* can affect infants by contaminated bottles, nipples, scoops, and other utensils not being properly cleaned as well as hands not being washed properly. Hospitals and other healthcare facilities, such as day cares, are at greater risks due to a high volume of infants and infant formula used. If not cared properly, children who are breastfed and drink from pre-pumped milk could also be infected with *C. sakazakii* if cross-contamination occurs from improperly sanitized breast milk pumps [41].

### 3.3. Importance of Breastfeeding in Prevention of C. sakazakii Infections

Both Centers for Disease Control and Prevention (CDC) as well as the World Health Organization (WHO) indicate the leading preventive measure for *C. sakazakii* infection in infants is breastfeeding. According to the CDC breastfeeding data, 82.5% of mothers at the beginning of birth breastfeed alone and this percentage decreased to only 24.9% of mothers who continue to breastfeed with no infant formula for their infants after six months of age. Additionally, 55.3% of mothers continue to breastfeed as well as use infant formula after the infant was six months of age [47]. One main reason parents choose infant formula is due to malnutrition in their child. Breastfeeding is a natural way to prevent undernutrition as well as infectious conditions, such as diarrhea and pneumonia in infants [48]. In addition, epidemiological study indicates mothers who breastfeed might have decreased chances of type II diabetes, depression and breast and ovarian cancers later on in life [48]. Ways to stretch breastmilk is to use breast pumps and pre-storing milk in the freezer. This is great for busy mothers and those who have others watching their child. To ensure safety with pre-pumped breastmilk, the breast pump should be washed and sterilized after every use [48]. Getting the correct storage container such as a freezer bag is a great way to store breast milk. Labeling and dating is also good practice to know exactly when the milk was expressed. Good hygiene is also recommended to safely store breastmilk. One of the biggest problems with breastfeeding is the support from family, friends, co-workers, and the hospital. For co-workers and in a workplace setting, paid maternity leaves is required. Designating an area for breastfeeding if the mother decides to bring the child to work is highly recommended [49]. Despite demonstrated health benefits, and although a low rate of breastfeeding adds as high as $2.2 billion a year to medical costs in the United States, according to Centers for Disease Control and Prevention, most US hospital do not fully support breastfeeding [50]. Once the mother starts on durations of infant formula, it is harder for the mother to get back into a habit of breastfeeding exclusively. At the six month mark, infants are ready for solid food and can start eating products such as infant cereal and pureed vegetables [51]. Mothers with HIV who want to breastfeed are recommended to take antiretroviral treatment to reduce the chances of transferring the infections to their child. Another option is obtaining donated or purchasing expressed breastmilk from mothers who are willing to help. There is a process that the donating mothers must go through. First, an application has to be filled out and sent to the company one wish to donate their milk for medical confirmations. Second, a test kit will be sent to the mother and a series of tests with their milk have to be conducted. If screening requirements are passed, then a nurse will be invited into the home for blood testing and other testings that may require a nurse’s assistant. If the medical tests are passed, then the mother could begin to label, filling, freezing, and packaging their breast milk. When arrived at the company, it is important to have the breastmilk still frozen after transportation. Further tests are then conducted then the milk is stored in the freezer until use [52].

### 3.4. Preventive Measures during the Use of Infant Formula for Reducing Risk of C. sakazakii Infections

As stated before, during powdered infant formula manufacturing, companies must add some of the nutrients (vitamins, minerals, amino acids, and fatty acids) after sterilization to avoid denaturation, thus, powdered infant formula is a non-sterile product. This is where pathogenic bacteria, such as *C. sakazakii* can be introduced into the infant formula. Typically microbiological sampling is conducted in a manufacturing facility, however *C. sakazakii* has the chance of presence in small quantities of a large batch of the powdered infant formula with heterogeneous distribution. Thus, sampling alone does not necessarily assure the safety of the product. Since powdered infant formula is the cheapest and most abundant form of infant formula, it is the most bought and used by parents, hospitals, care centers, and other healthcare facilities [45]. For the preparation of this product, hands must be washed before, during, and after preparing powdered infant formula mix [53]. If soap and clean water are not available, then using a sanitizer that is more than at least 60% alcohol can be used as a replacement [53]. The World Health Organization has specific instructions for the preparation of powdered infant formula in care settings and in the home [54]. Since in care settings there are high volumes of infants and many packages of powdered infant formulas might be in use, the recommendations are on a larger scale than those in the home setting. When preparing infant formula, the preparation area would need to be cleaned and disinfected due to the potential presence of microbial pathogens on abiotic surfaces including *C. sakazakii* [54]. Next is to boil a generous amount of water, relevant to the number of infants going to be fed. This eliminates all bacterial microorganisms in planktonic form. Feeding bottles are also not sterile and must be boiled before use. Microwaving should not be used when preparing powdered infant formula. Then the water would need to be cooled slightly but not under 70 °C, then the proper amount of formula could be added to clean and sterilized feeding cups or bottles. If using a larger container, it should not exceed over 1 L [54]. For mixing, bottles can be shaken to fully mix the water and powdered infant formula, feeding cups can be stirred with a pre-sterilized spoon, and large containers can be stored with spoons but has to be distributed to its respective containers immediately to avoid scalds for infants [54]. In a care setting, bottles then would need to be labeled with infant’s name, type of formula, or ID, as well as the time and date prepared and the preparer’s name to assure traceability is possible in case of contamination occurrence. If intending to prepare powdered infant formula for later use, it is best to prepare new batches of feeding every time and to eliminate leftovers. Leftover feed is suggested to be thrown away and especially not to be used if stored more than two hours at room temperature [54]. However, it can be stored in a sealed container in the refrigerator for up to 24 h. To re-warm stored infant formula, a separate container filled with boiled water could be used to place the bottle or feeding cup inside to evenly warm up the reconstituted product without damaging the micronutrients. Recommendation for the home setting is similar to the care setting facilities on a smaller scale [55]. Hands must be washed properly, equipment being used for preparation must be cleaned with hot soapy water and be scrubbed inside and outside of the bottle, then rinsed thoroughly. Sterilizing equipment is also recommended to eliminate pathogenic microorganisms. For sterilization, a large pan with water could be used to place equipment inside of the pan filled with water, covering the pan, and to bring the content to a boil. Similar to a recommendation for the care setting, the recommended amount could be poured to water with the temperature not under 70 °C. The bottles then could be shaken or swirled gently. The bottle content temperature could be tested on the skin to ensure that it is warm and not too hot for feeding the infant. If it hasn’t been used in two hours at room temperature or more, prepared infant formula would need to be discarded [55]. When transporting the prepared milk, the product would need to be kept cold during transportation to slow down or stop the multiplication of potentially pathogenic bacteria. In other cases, where there is no access to boiling water or preparation of powdered infant formula cannot be made at the time, it is recommended to use ready-to-feed infant formula. It is sterile from the processing manufacturing and ready to use without any additional ingredients [45].

## 4. Fate and Multiplication of *C. sakazakii* on Biotic Surfaces

As previously discussed, *C. sakazakii* is a Gram-negative, facultatively anaerobic, non-sporulating, motile rod-shaped bacterium and is a member of the Enterobacteriaceae family. *C. sakazakii* multiplies between temperatures of 6–45 °C with an optimum temperature of 37–43 °C. It could survive and/or proliferate in water activity levels ranging from 0.30 to 0.83 [9]. The bacterium is widely associated with powdered infant formula from many outbreaks [19,22,26] as previously articulated, thus, powdered infant formula is the essential biotic reservoir of public health concern for *C. sakazakii*. According to the United States Department of Agriculture National Nutrient Database [56], a typical powdered infant formula has a protein content of 1.02g/scoop, total lipid of 2.35 g/scoop, carbohydrates 4.86 g/scoop, seven minerals: calcium, iron, magnesium, phosphorus, potassium, sodium, and zinc; and 13 vitamins: Vitamin C, B-6, B-12, A (RAE), A (IU), E, D (D2 + D3), K, thiamin, riboflavin, niacin, and folate [56]. Since all powdered infant formula has to follow the same guidelines for nutritional value by the Food and Drug Administration, all powdered infant formulas are nearly identical in formulation unless specified for premature or low iron infants [9]. 

### 4.1. Fate and Multiplication of C. sakazakii as Affected by Temperature

From the 1980s to the present time, there are several studies on determining the fate and multiplication of *C. sakazakii*. Strains of *C. sakazakii* isolated from clinical, food, and/or environment could be used for various microbiological challenge studies. In 1980, researchers delineated the multiplication rate of the pathogen using strains sent to the CDC from patients and one from an unopened can of dried milk. Of the 57 strains used in their experiment, all grew at 25, 36, and 45 °C and 50 of the strains grew at 47 °C [57]. It was noted that none of the strains grew at 4 or 50 °C. The pathogen grew in presence of d-glucose without added nutrients such as vitamins, minerals, or amino acids. Additionally, the researchers grew the pathogen in aerobic and anaerobic environments. The strains were monitored on tryptic soy agar and all strains grew on the agar at 36 °C after 24 h. The strains produced bright yellow colonies on the agar, known as a typical characteristic of *C. sakazakii* today. It is noteworthy that this pigmentation alone should not be considered as a species criterion [5].

The growth of *C. sakazakii* in broth was monitored in tryptic soy broth and all strains of *C. sakazakii* produced large amounts of sediments. The biochemical reactions of the 57 strains were also conducted [57].

### 4.2. Survival Rate

A study published in 1997 shows the survival and multiplication of *C. sakazakii* in powdered infant formula and on Brain Heart Infusion (BHI) medium, as well as the incidence of *C. sakazakii* being present in a Canadian supermarket purchase of one of the popular powdered infant formula brands [58]. Ten strains of *C. sakazakii*, five clinical and five food isolates, were used for this experiment. The minimum growth temperature was 5.5 °C for one clinical strain and two food strains on BHI broth, the study also indicated that none of the remaining strains grew under 5.5 °C. In powdered infant formula, the lag time at 10 °C for the formula that contained the food strain (19 h) was less than the formula with the clinical strain (47 h). For generation time at 10 °C, it ranged from 4.18 to 5.52 h with the formula inoculated with the clinical strain exhibiting the longer time. At 23 °C, the mean generation time was 0.67 h. With generation time of 0.67 h at room temperature, it is evident that leaving reconstituted infant formula on countertops or traveling with it without proper refrigeration could potentially increase the risk of *C. sakazakii* infection. After testing 120 powdered infant formulas in a Canadian supermarket, the researchers also observed a 6.7% presence of *C. sakazakii* in the market [58].

In a later study, published in 2006, the objective was to prevent the multiplication of *C. sakazakii* in media and powdered infant formula using bacteriophages [59]. Bacteriophages are viruses that infect bacteria. There are now exploratory studies proposing the use of bacteriophages to control foodborne bacterial pathogens and spoilage bacteria in live animals, meat, dairy products, seafood, and fresh produce [35]. A total of six *C. sakazakii* strains were used, one clinical and five food isolates, and were incubated in powdered infant formula and BHI at 12, 24, and 37 °C. The *C. sakazakii* bacteriophages were prepared from an environmental water sample (centrifuged and sterilized) with an equal amount of BHI, mixed and incubated overnight at 24 °C. Then, against the six *C. sakazakii* strains, five bacteriophages were used. For the powdered infant formula, *C. sakazakii* bacteriophages at 37 °C, the clinical strain showed a decrease in multiplication starting at 2 h and at 24 °C. At 12 °C, it was not significantly different compared to the control. For the food strain in powdered infant formula, the results were similar at 12 °C as for the clinical strain but at 24 and 37 °C they were both reduced by one log immediately at 2 h and one strain continued to be at its detection limit for the duration of 2–10 h. This exploratory study showed with the correct temperature and bacteriophage concentration, the inactivation of *C. sakazakii* could be achieved. The most effective reduction was at the highest bacteriophage concentration of 10^9^ at any incubation temperatures of 12, 24, or 37 °C [35].

### 4.3. Water Activity

Water activity (A_w_) is the measurement associated with the availability of water in biological setting and relates to water presence in the food in free form [9]. Water activity could range from 0.1 to 0.99 in foods. In powdered infant formula, the A_w_ can start at 0.20 depending on the added nutrients and differing for soy or milk based products. *C. sakazakii* could survive in powdered infant formula for two years at low A_w_ [60,61]. In a study by Joshua B. Gurtler et al., an experiment was conducted to see the survival rate of *C. sakazakii* in soy-based and milk-based powdered infant formulas at various A_w_ ranging from 0.25 to 0.86 at 4, 21, and 30 °C for 12 months [62]. There were 10 *C. sakazakii* strains used: five clinical, four food, and one environmental isolate; a total of six powdered infant formulas were used (four milk-based and two soy-based) and water activity divided into two categories of low A_w_ of 0.25–0.50 and high A_w_ 0.43–0.86. The A_w_ was adjusted by adding amounts of saturated salt solutions to lower or raise the A_w_. In high A_w_ infant formula, the rate of inactivation of *C. sakazakii* increased as the storage temperature increased. This study notes that augmenting the pathogen inactivation is possible with the increase of A_w_ and temperature during storage. This study also states that the clinical strains survived longer than the food strains in powdered infant formulas at high A_w_ [62].

### 4.4. Thermal Inactivation

Thermal inactivation validation studies are conducted to determine the highest temperature a pathogen can withstand. When heat treatment is applied, the decimal reduction time, D-value, could be calculated. If at a specific temperature the pathogen is being reduced, it could be reported in the context of 1 log reduction or 90% deactivation. This is where one could mathematically assimilate if the pathogen is being reduced over a course of treatment. A study published in 2003 delineates that *C. sakazakii* is not thermotolerant, but resistant to osmotic stress and drying [63]. The temperatures used for heat treatment for the 22 *C. sakazakii* strains were at 53, 54, 56, or 58 °C at different time intervals in a water bath. After heat treatments, the samples were immediately cooled in iced water for 1 min and then enumerated to see the heat treatment results. For the preparation of dry stressed strains, plates of *C. sakazakii* were kept without a lid in a 25 °C incubator for air-drying. This was monitored for 46 days for *C. sakazakii* survival after air-drying. All 22 *C. sakazakii* strains multiplied in 47 °C BHI broth. For heat resistant phenotype, the D-value at 58 °C in phosphate buffer pH of 7 had a mean value of 0.48 min and in reconstituted infant formula it did not have a significant difference. For resistance to dry stress, *C. sakazakii* at 25 °C was decreased by 1–1.5 log unit after 46 days. This study concludes that relative to many other members of the Enterobacteriaceae family, *C. sakazakii* appears to be more resistant to osmotic and dry stress [63].

## 5. Survival and Biofilm Formation on Abiotic Surfaces

*C. sakazakii* has not only been reported to survive on various biotic surfaces, such as infant formulas, fruits, vegetables, or human intestines, but also on abiotic surfaces, such as stainless steel and polyester plastic. The pathogen is also capable of forming a sessile community of bacterial biofilms on both biotic and abiotic environments. Specifically, the bacterium is capable of forming a polyanionic extracellular polysaccharide also known as ESP [9]. Study of Kumar et al. delineates the process of biofilm formation into three parts: conditioning of a surface, adhesion of cells, and formation of microcolonies. Within the food industry, surfaces of equipment can be coated with nutrients from the food product enhancing the biofilm formation of the pathogen [64]. Adhesion is in two stages, reversible adhesion followed by an irreversible adhesion. The former starts with weak interactions within the bacterial cells and the substratum. The ability to maintain levels of the van der Walls attraction forces, electrostatic forces and hydrophobic interactions determines the next stage to irreversible adhesion. Irreversible adhesion is a repulsive force that prevents the bacterial cells and the biofilm community from disassociation from the surface. The next stage will be formation of a microcolony where the irreversibly-attached cells are dividing. This formation of microcolony begins the formation of a visual thick layer of organisms formed on abiotic surfaces. ESP is also being produced for a firmer attachment to the abiotic surfaces [64].

One of the main surfaces of concern for *C. sakazakii* infection is the feeding tubes of neonates after being fed reconstituted powdered infant formula. In a study by Hurrell et al., biofilm formation was conducted on enteral feeding tubes [65]. Twelve strains of *C. sakazakii* were used in this study from various patients, infant formulas, enteral feeding tubes, and raw materials. The tubing materials that were selected were polyvinyl chloride (PVC) and polyurethane (PU). The pathogen was inoculated into powdered infant formula and incubated overnight at 37 °C and then diluted after 18 h. This cocktail was then aseptically syringed through the tubes to observe the biofilm formation in two-hour intervals. As a result, 5 strains of *C. sakazakii* produced biofilm mass with a density of 10^8^ to 10^10^ CFU/mL. One strain was from a patient, three from powdered infant formula, and one from an enteral feeding tube. The doubling time for the *C. sakazakii* strains isolated from the enteral feeding tubes was between 22–27 min [65].

Another study with biofilm formation on enteral feeding tubes utilized clinical, food, and environmental isolates [66]. Sterile feeding tubes were used and the tubes were inoculated with *C. sakazakii*. For biofilm formation, the tubes were incubated at 4 °C for 24 h and then rinsed and submerged in phosphate-buffered saline. Next, it was divided into two groups monitored for a course of 10 days: 2, 4, 6, 8, and 10 days at 12 °C and 1, 2, 4, 6, 8, and 10 days at 25 °C. The results show attachment and growth of sessile cells were similar among the strains used. The multiplication rates were much higher at 25 °C than 12 °C by 3–4 logs, however, in both temperatures the rate was constant over the course of ten days showing no significant differences. Along with feeding tubes, other plastic surfaces are crucial such as bottles and other equipment [66].

In another study, biofilm formation on plastic surfaces was investigated. Four strains of *C. sakazakii* were used from the American Type Culture Collection (ATCC). For biofilm formation, plastic microtiter plates were used [67]. The plates were inoculated with *C. sakazakii* and incubated for 24 h at 37 °C. The plates were rinsed with distilled water and then submerged with methanol for 15 min. Crystal violet was used to visualize pathogenic growth on the plastic microtiter plates. The results show that out of the four strains used in this experiment, not all produced biofilms in artificial media alone. For biofilm formation on plastic surfaces, 13.9% populated in Brain Heart Infusion and tryptic soy broth mixture and 6.9% populated in nutrient broth [67].

## 6. Current Decontamination Strategies

### 6.1. Disinfectants to Eliminate Biofilm Attachment

Various validated antimicrobial agents could be used for inactivation of *C. sakazakii* from biotic surfaces associated with production and preparation of infant formula as well as feeding tubes and equipment in hospitals [9]. Just like decontamination of other microbial pathogens, cleaning alone may lead to modest reductions in removal of a pathogen [68]. To ensure that pathogens are eliminated or reduced to a microbiologically acceptable level, validated sanitation is recommended directly after cleaning. Sanitation could be achieved by physical means such as heated water, UV radiation, or could be achieved using chemical agents such as chlorine-based, iodophors, quaternary ammonium compound sanitizers, and hydrogen peroxide [9].

In general, chlorine-based sanitizers could be very effective against planktonic cells of bacteria, yeast, and molds. Similarly, iodophors are effective against Gram-positive and Gram-negative bacteria, bacterial spores, viruses, and fungi. Quaternary ammonium compounds are also known as an efficacious sanitizer due to their ability to clean and to sanitize surfaces against an array of microorganisms at acidic pH and higher temperatures. Hydrogen peroxide is also a very effective antimicrobial against planktonic bacterial cells, spores, and viruses [9]. 

In the study of Kim et al., the objective was to see the effectiveness of disinfectants in eliminating *C. sakazakii* [69]. Due to widespread use in infant formula preparation areas, laboratories and hospitals, food services, and child day care settings, quaternary ammonium and phenolic disinfectants were evaluated in their study. Overall, 13 disinfectants were studied in their investigation from various suppliers against biofilm formation of *C. sakazakii* on stainless steel. This experiment was evaluated with two strains of *C. sakazakii* studying biofilm formation on stainless steel at days 6 and 12 as well as the treatment times of 0, 1, 5, and 10 min for submersion in disinfectants. For quaternary ammonium compound-based disinfectant, *C. sakazakii* was reduced to less than 0.30 log CFU/mL within 1 min of submerging into the sanitizer. The quaternary ammonium compound-based sanitizer applied as a spray product were, however, showed only modest reductions. Another effective sanitizer, peroxyacetic acid/hydrogen peroxide, resulted in a log reduction of >2.4 log CFU when applied for 10 min. This study shows that quaternary ammonium compound-based and peroxyacetic acid sanitizers could be very effective for inactivation of the pathogen in planktonic and biofilm stages if used at optimized conditions. Our recent studies, however, indicate that previously validated sanitizers against planktonic cells might not be able to completely eliminate one- and two-week mature biofilms from stainless steel [70,71]. Thus, commercial adoption of a cleaning and sanitizing program requires careful consideration of existing literature and conduct of microbiological validation studies against sessile and planktonic cells for specific intrinsic and extrinsic conditions of a product and processing area.

### 6.2. Thermal Inactivation

Thermal inactivation could be an efficacious method for decontamination of *C. sakazakii* from biotic and abiotic surfaces. As briefly introduced in Section 6.1, elevated heat could be used as a physical mean for decontamination of significant surfaces associated with the production, manufacturing, and preparation of infant formula. It could be utilized as a processing aid for assuring the safety of the product, through high temperature short time (HTST) pasteurization. Decontamination of *C. sakazakii* could typically be achieved at temperatures 70 °C or higher [72]. In a study, twelve strains were used in reconstituted infant formula prepared based on manufacture’s instruction, then 15 mL of infant formula was inoculated with 1.5 mL of *C. sakazakii* and injected into the heating coil apparatus at set temperatures of 58 °C [72]. After being in contact with the controlled heat, the samples were immediately placed on ice to discontinue further heat decontamination. The temperature 58 °C was the set point for z- and D-values to be calculated followed by treatments at temperatures of 56, 60, 65, and 70 °C. The study concluded that to fully inactivate a heat-resistant strain of *C. sakazakii*, temperatures of 70 °C or greater are needed. This elevated temperature could lead to nutrient loss and unwanted changes in organoleptic properties of infant formula [72], thus preservation of heat labile micronutrients is considered as a major curtailment for successful implementation of sterilization or pasteurization of powdered infant formula from *C. sakazakii* in commercial manufacturing. This indicates the need for innovative and emerging technologies [70].

### 6.3. High-Pressure Processing

High-pressure processing (HPP) or high hydrostatic pressure (HHP), is a non-thermal method involving pressurization of a packaged food in a water-filled closed chamber, for a short duration to inactivate microorganisms [9]. The technology popularity in private industry is gaining momentum in recent years with purchase rates reaching nearly 200 units around the world [73]. Beneficiary aspects of using HPP is the preservation of the color, flavor, freshness and physical properties of foods with minimal damages to nutritional values [74]. The HPP machines can have low to high pressures ranging from <100 to 1000 MPa [75]. According to the National Advisory Committee on Microbiological Criteria for Foods, pasteurization that had been traditionally known as a heat-based intervention is now redefined and HPP is a part of pasteurization definition as a non-thermal pasteurization method [73].

In a study by Arroyo et al., four strains of *C. sakazakii* were used, exposed to elevated hydrostatic pressures ranging from 200 to 600 MPa and for 0 to 10 min [76]. This study also investigated four food vehicles for inoculation: orange juice, chicken soup, vegetable soup, and rehydrated powdered milk. In all food vehicles, the most pressure-resistant strain showed around 3 log reductions at 500 MPa, reaching the study detection limit. The study results indicated utilization of elevated hydrostatic pressure could eliminate the pathogen from biotic surfaces, it also articulates that various isolates of *C. sakazakii* could exhibit considerably different sensitivity to hydrostatic pressure [76]. Our recent studies also exhibit that various phenotypes of *C. sakazakii*, such as rifampicin-resistant variants and pressure-stressed isolates could be inactivated by over 5 logs, using elevated hydrostatic pressure of up to 380 MPa, in rates that are comparable with wild-type isolates [70]. Effects of hydrostatic pressure on retention of heat liable micro and macronutrients of infant formula is currently a knowledge gap of literature and could be considered as the main curtailment for widespread adoption of high-pressure processing in infant formula manufacturing.

It is noteworthy that, in addition to pressure-based interventions, an array of emerging and re-emerging technologies such as utilization of ohmic, microwave, radio frequency, ultrasonic, or infrared heat; pulsed X-rays; pulsed electric field; and oscillating magnetic field could potentially exhibit promising applications for microbiologically efficacious and economically feasible treatment of infant formula against planktonic cells and biofilms of *C. sakazakii*. These exploratory applications require microbial challenge and safety validation studies as well as feasibility assessments. As an example, mild temperature of up to 50 ºC coupled with an ultrasonic treatment at amplitude of up to 61 μm, could yield microbiological reductions comparable to traditional heat treatments for inactivation of *C. sakazakii* [77].

## 7. Conclusions

There have been a few recent outbreaks and sporadic cases of *C. sakazakii* infections in the country and around the world associated with infant mortality and morbidity. This is almost certainly an underestimation of the public health burden of the pathogen since unlike the vast majority of main foodborne pathogens, *C. sakazakii* infections are not currently a reportable disease in nearly all states. Strict regulatory standards for nutritional quality of powdered infant formula and heat sensitivity of micronutrient additives of the product lead to the need to addition of the heat-labile ingredients of the formula after heat treatment that creates a potential route of contamination of the product with this bacterium. The ability of this bacterium to survive osmotic stress and low water activity environments for as long as two years, further provides an acceleration in the likelihood of this disease occurrence in the vulnerable population. The *C. sakazakii* has the potential to survive and persist on various biotic and abiotic surfaces such as preparation area in healthcare facilities and form biofilm communities that are more resistant to antimicrobial interventions. These characteristics add another layer of complexity for elimination and prevention of *C. sakazakii* from the manufacturing facilities, hospitals, and domestic environments. Considering the nature of contamination of products in food manufacturing that is mostly heterogeneous in nature as a fraction of larger batches, sampling alone could not assure the safety of infant formula and could lead to false sense of security for manufacturers. Use of supplier’s chain verification programs, relying on food safety management system such as those articulated in Food Safety Modernization Act or Hazard Analysis Critical Control Point-based regulations, as well as the use of emerging and validated technologies such as utilization of elevated hydrostatic pressure could assure the safety of the infants and powdered infant formula products. Following the articulated recommendations for the preparation of reconstituted infant formula in healthcare and domestic settings, and reliance on breastfeeding when medically possible are the main preventive approaches that could be implemented by parents and healthcare providers to minimize the risk of infection with this opportunistic bacterium.
